# Hepatoprotective Potential of Malaysian Medicinal Plants: A Review on Phytochemicals, Oxidative Stress, and Antioxidant Mechanisms

**DOI:** 10.3390/molecules27051533

**Published:** 2022-02-24

**Authors:** Balu Alagar Venmathi Maran, Mohammad Iqbal, Prakash Gangadaran, Byeong-Cheol Ahn, Pasupuleti Visweswara Rao, Muhammad Dawood Shah

**Affiliations:** 1 Borneo Marine Research Institute, Universiti Malaysia Sabah, Kota Kinabalu 88400, Sabah, Malaysia; bavmaran@ums.edu.my; 2 Biotechnology Research Institute, Universiti Malaysia Sabah, Kota Kinabalu 88400, Sabah, Malaysia; miqbal@ums.edu.my; 3 BK21 FOUR KNU Convergence Educational Program of Biomedical Sciences for Creative Future Talents, Department of Biomedical Sciences, School of Medicine, Kyungpook National University, Daegu 41944, Korea; prakashg@knu.ac.kr (P.G.); abc2000@knu.ac.kr (B.-C.A.); 4 Department of Nuclear Medicine, School of Medicine, Kyungpook National University, Kyungpook National University Hospital, Daegu 41944, Korea; 5 Department of Biomedical Sciences and Therapeutics, Faculty of Medicine and Health Sciences, Universiti Malaysia Sabah, Kota Kinabalu 88400, Sabah, Malaysia; pvrao@ums.edu.my; 6 Department of Biochemistry, Faculty of Medicine and Health Sciences, Abdurrab University, Pekanbaru 28292, Riau, Indonesia; 7 Centre for International Collaboration and Research, Reva University, Rukmini Knowledge Park, Kattigenahalli, Yelahanka, Bangalore 560064, Karnataka, India

**Keywords:** medicinal plants, oxidative stress, phytochemicals, hepatoprotective, carbon tetrachloride

## Abstract

Hepatotoxicity is a major global public health concern. Despite advances in modern medicine, the demerits of chemically prepared drugs outweigh their merits. In addition, the treatment of liver diseases based on modern medical principles has been found to produce several undesired side effects. Therefore, the exploration of medicinal plants has gained worldwide attention for treating various diseases, including liver diseases, owing to their potential efficacy and cost effectiveness. Several plants, including *Andrographis paniculata*, *Bauhinia purpurea*, *Commelina nudiflora*, *Dillenia suffruticosa*, *Elaeis guineensis*, *Lygodium microphyllum*, and *Nephrolepis biserrata*, have been reported with hepatoprotection. Moreover, these plants have been shown to play a vital role in ameliorating cellular damage because they contain several phytochemicals, including alkaloids, saponins, flavonoids, tannins, terpenoids, steroids, polyphenols, and diterpenoid lactones. The following antioxidant, anti-inflammatory, immunomodulatory, and hepatoprotective compounds have been found in these plants: andrographolide, rosmarinic acid, phenol, eugenol, 9,12-octadecadienoic, n-hexadecanoic acid, dihydroxy dimethoxy flavone, sitosterol, demethoxycurcumin, quercetin, linoleic acid, stigmasterol, kojic acid, indole-2-one, α-terpinol, linalool, kaempferol, catechin, ellagic acid, and oleanolic acid. This paper aimed to provide an in-depth review of in vivo studies on Malaysian medicinal plants possessing hepatoprotective properties, phytochemical ingredients, and antioxidant mechanisms, with an emphasis on the species proven particularly useful for treating hepatic disorders.

## 1. Introduction

The liver plays an essential role in the regulation of physiological functions [1]. It is involved in almost all biochemical pathways responsible for growth, immunity, nutrient supply, energy provision, and reproduction. A major function of the liver is the metabolism of carbohydrates, proteins, and fats [1,2]. It also plays a vital role in the storage of glycogen, hormones, vitamins, iron, minerals, and many other substances [3]. Furthermore, it represents the primary destination for toxins absorbed from the intestinal tract and is involved in the breakdown and elimination of toxins, including drugs and other foreign chemical substances [4].

The liver detoxifies and transforms numerous toxic substances so that they can be discharged through the kidneys for urine removal or into bile for colon clearance [5]. The liver also plays a fundamental role in the metabolism of various drugs. Once in the liver, drugs are converted by enzymes into active metabolites or inactive forms. Drugs are primarily metabolized in the liver by a group of enzymes known as cytochrome P-450 [4,5]. Therefore, the liver is highly vulnerable to toxic compounds because of its complex functions.

Hepatic illnesses result in the mortality of thousands of people all over the world annually. Around 2 million people die each year from liver disease (1 million from cirrhosis complications and 1 million from viral hepatitis and hepatocellular cancer) [6]. Despite significant developments in modern medicine, no fully effective medications exist to promote hepatic function, provide total organ protection, or assist in the regeneration of hepatocytes [7]. Steroids, antiviral drugs, and immunizations are commonly used to treat and prevent liver illnesses; nevertheless, they are not only expensive, but they also have the potential to induce side effects [8,9,10]. Several studies have reported that medicinal plants and their phytochemical compounds help protect against various hepatic disorders [8,11,12,13]. Hence, considerable attention has been devoted to the identification of medicinal plants with antioxidant, preventive, and therapeutic potential against hepatic diseases.

The review article aimed to provide an in-depth review of in vivo studies on Malaysian medicinal plants possessing hepatoprotective properties, phytochemical ingredients, and antioxidant mechanisms, with an emphasis on the species proven particularly useful for treating hepatic disorders.

## 2. Hepatotoxicity and Liver Diseases

Various pathological features are associated with liver diseases, including noninflammatory (hepatosis), inflammatory (acute or chronic hepatitis), and tumorous (hepatic adenoma or hepatocellular carcinoma) disorders [14,15]. Liver injury can be caused by different types of chemically prepared drugs, e.g., paracetamol (PCM), fluconazole, amoxicillin, diclofenac, ciprofloxacin, oral contraceptives, chlorpromazine, and antitubercular drugs. These drugs can cause fulminant hepatitis, liver necrosis, benign neoplasm, and hepatic vein occlusion. Toxic compounds, such as alcohol, aflatoxin, and carbon tetrachloride (CCl_4_), can also cause liver toxicity [4,16,17].

Every year, approximately 2 million people die from viral hepatitis, liver cirrhosis, and hepatocellular carcinoma [6]. Hepatic cancer is the 16th leading cause of cancer-related death in the world [6,18,19,20]. Excessive alcohol consumption, obesity, viral infections, and drugs are all important factors in the development and progression of liver disease. Approximately 2 billion people consume alcohol globally, >75 million of whom are diagnosed with alcohol use disorders and remain at risk for alcohol-associated hepatic injuries [21,22]. Approximately 2 million adults and >400 million people suffer from obesity and diabetes, respectively, both of which further increase the risk of hepatic disorders, such as nonalcoholic fatty liver disease and hepatocellular carcinoma [23,24]. Acute hepatitis, caused by hepatitis B and C viral infections, further increases the risk of hepatic injuries [24,25]. At present, Malaysia is faced with the heavy burden (both medical and financial) of ongoing liver diseases, which will continue at least for the next 20–30 years [26]. In Malaysia, hepatitis B infection represents the dominant cause of liver cirrhosis and hepatocellular carcinoma in all major races, including Malay, Chinese, and Indian [27,28].

Exposure to the above-mentioned compounds and hepatotoxic agents results in oxidative stress through abundant reactive oxygen species (ROS) production. Overproduction of ROS increases lipid peroxidation and causes oxidative damage to hepatic cells, resulting in hepatic steatosis, chronic hepatitis, cirrhosis, and hepatocellular carcinoma [29,30].

## 3. Oxidative Stress and ROS

Oxidative stress is a phenomenon caused by the imbalance between free radicals and antioxidants in our bodies [31]. It has been linked to more than 200 diseases [32].

ROS are highly unstable molecules with only one electron in their outer shell, and they play a key role in tissue damage. Nitric oxide (NO**^•^**), superoxide anion (^•^O_2_^−^), hydroxyl radical (**^•^**OH), and hypochlorous acid (HOCl) are the most common examples [33]. They damage the cells by interacting with macromolecules such as proteins, lipids, and DNA. The effects of ROS on proteins are largely mediated by the initial modification of cysteine, methionine, histidine, tyrosine, and tryptophan residues, which triggers degradation and conformational changes [34,35].

Lipids—more specifically, phospholipids—are another essential component of the cell membranes and organelles (e.g., nucleus and mitochondria). ROS attack polyunsaturated fatty acids in the membranes and induce cell death through the lipid peroxidation process, whereby reactive aldehydes (malondialdehyde [MDA] and 4-hydroxynonenal [HNE])—which further create protein cross-linkages—are produced, intracellular reduced glutathione (GSH) is depleted, peroxide generation is promoted, the epidermal growth factor receptor is activated, and fibronectin formation is stimulated [34,36]. HNE and MDA are considered the cytotoxic second messengers of oxidative stress signaling and are therefore important biomarkers for the investigation of oxidative injuries [37].

ROS can cause lasting damage to DNA molecules, including strand disruption, base modification (e.g., deoxyguanosine to 8-hydroxy-2′-deoxyguanosine), nucleotide deletion, various alterations in the organic bases of nucleotides, and cross-linking with proteins [38]. Moreover, ROS can oxidize both nuclear and mitochondrial DNA molecules, with the latter being more (10–20 times) vulnerable to oxidative injury than the former [39]. The majority of these DNA changes are linked to cancer, ageing, neurodegenerative diseases, autoimmune disorders, and cardiovascular illnesses [34]. They also cause adult/acute respiratory distress syndrome, disseminated intravascular coagulation, sepsis, chronic gastrointestinal inflammation, and hepatic disorders [40].

ROS also regulate various types of kinases and transcription factors (including nuclear factor-kappa B), which is related to the expression of proinflammatory markers (such as tumor necrosis factor-alpha [TNF-α], interleukin 6 [IL6], and prostaglandin E2 [PGE2]) [41].

### Defense Mechanisms against ROS

**Figure 1:**
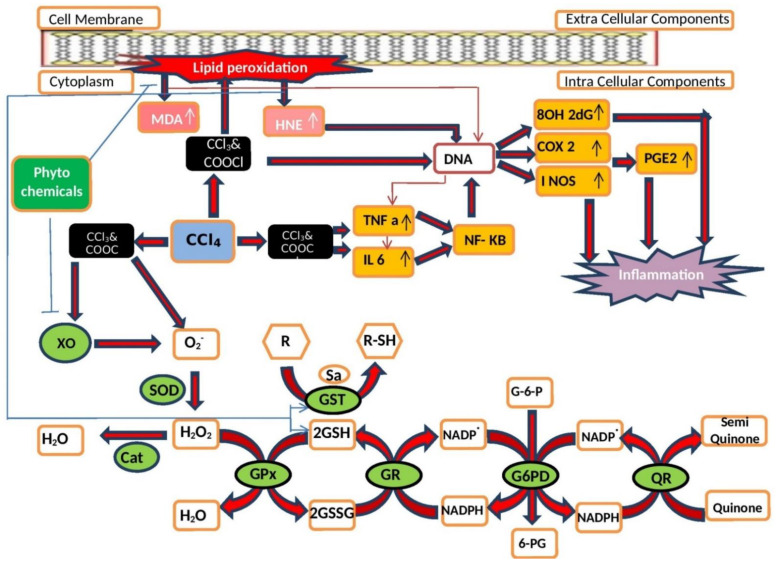
Effects of carbon tetrachloride (CCl_4_)-induced ROS on the defense mechanisms of antioxidant enzymes and phytochemical compounds with antioxidant properties. The antioxidant enzymes xanthine oxidase (XO), superoxide dismutase (SOD), catalase (CAT), glutathione peroxidase (GP), glutathione reductase (GR), glucose 6 phosphate dehydrogenase (G6PD), glutathione S-transferase (GST), and quinone reductase (QR) along with phytochemical compounds can reduce the harmful effects of ROS by decreasing the extent of malondialdehyde (MDA) production, elevating the levels of reduced glutathione (GSH), and reducing the overproduction of 4-hydroxynonenal (HNE) protein adducts and 8-hydroxy-2′-deoxyguanosine (8OHdG) as well as by overexpressing tumor necrosis factor-alpha (TNF-α), interleukin 6 (IL6), and prostaglandin E2 (PGE2).

Both enzymatic and nonenzymatic mechanisms are involved in preventing or minimizing the deleterious effects of ROS [42]. Antioxidant enzymes constitute an important part of the antioxidant defense system; that is, they play a crucial role in the elimination of ROS. Enzymes involved in the direct detoxification of ROS are glutathione peroxidases (GPs), catalase (CAT), superoxide dismutases (SODs), glutathione S-transferase (GST), and quinone reductase (QR). GPs, CAT, and SODs are known as primary enzymes and GST and QR are secondary enzymes. SODs help in the removal of toxic superoxide radicals, whereas CAT and GPs both assist in the elimination of hydrogen peroxides [43]. Nicotinamide adenine dinucleotide phosphate (NADPH) and GSH are nonenzymatic antioxidants and part of the antioxidant defense system. They have a substantial role in the removal of ROS. To be more precise, NADPH is engaged in the GP system, whereas ROS produced during the respiratory chain in mitochondria are removed by GSH. In addition, other nonenzymatic antioxidants (phytochemical compounds), such as α-tocopherol (also known as vitamin E) and ascorbic acid (also called vitamin C), play integral roles in the detoxification of ROS [44]. The effects of ROS generated by the application of CCl_4_ on antioxidant enzymes and phytochemical compounds are indicated in Figure 1.

Oxidative stress is caused by the inhibition or lack of antioxidant enzymes, which can further damage and lyse cells. Antioxidant defense mechanisms are responsible for the prevention of free radical formation by oxidant scavenging, the transformation of harmful free radicals into less harmful substances, and the inhibition of the development of inflammatory mediators and secondary harmful metabolites. These defense systems work together to protect the body against the damaging effects of oxidative stress [45].

## 4. Medicinal Plants

Medicinal plants have been on earth since even before the appearance of human beings. They play a vital role in various biological activities. Thousands of various plant species are used globally in different human cultures for medicinal purposes. They contain bioactive compounds with antioxidant, anticancer, antimicrobial, anti-inflammatory, and hepatoprotective properties [46,47,48,49,50,51]. Many countries, including China, India, Malaysia, Korea, Egypt, and others, have developed their formulations with different medicinal plants and herbs [52]. According to the published data, nearly 25% of modern medicines have been developed from medicinal plants [53]. Approximately 65% of patients in the United States and Europe consume herbal medicines for liver diseases because of their wide availability, pharmacological activity, biochemical diversity, and fewer side effects than synthetic drugs [54].

Phytochemicals derived from medicinal plants serve to prevent illnesses and improve health and therefore have been widely examined to determine their efficacy and underlying mechanisms of action. According to studies, phytochemicals may lower the incidence of coronary heart disease by decreasing the oxidation of low-density lipoprotein cholesterol and enhancing the flexibility of arteries, detoxification of carcinogenic toxins, neutralization of free radicals, blockage of carcinogen-activating enzymes, and activation of carcinogen-detoxifying enzymes [55,56,57]. Phytochemicals are classified under two categories: primary metabolites, including chlorophylls, sugars, nucleic acids (purines and pyrimidines), and proteins, and secondary metabolites, including alkaloids, flavonoids, lignans, terpenes, saponins, curcumins, steroids, phenolics, and glucosides [58]. According to a literature review, phenolics are the most abundant and structurally varied plant phytochemical compounds that have been researched primarily for their potential activity against oxidative injuries responsible for degenerative disorders, such as cancer, inflammation-induced hepatotoxicity, and cardiovascular diseases [59,60].

### 4.1. Malaysian Medicinal Plants

Providing the habitat for thousands of species of medicinal plants, Malaysia ranks among the world’s 12 megadiverse countries; almost one-fourth of its tree flora is unique and not sighted elsewhere in the world [61,62,63]. In the states of Sabah and Sarawak in Malaysia, 2000 plant species with medicinal value have been discovered [64,65]. It has also been reported that in Peninsular Malaysia, 1200 species of higher plants have medicinal potential [65]. Traditional healers have utilized these plants to cure a variety of ailments, including hepatic disorders.

Sabah in Malaysia is rich in plant biodiversity and gifted with numerous medicinal plants, which are used by local people to treat various ailments [66]. These plants contain numerous compounds with antimicrobial, antimalarial, anticancer, antidiabetic, and hepatoprotective properties. Plant species collected from various parts of the Maliau Basin, Sabah, Malaysia, have been reported to include a broad range of active ingredients, such as steroids, alkaloids, saponins, and triterpenoids [66,67].

One of the medicinal plants used by the local people in Malaysia for treating various diseases is *Aloe vera*, which is locally known as Dihabuazo. The sticky sap obtained from its leaves is used to treat skin itches, cuts, burns, and stomach aches. This plant contains a variety of bioactive chemicals, including hydroxyanthraquinones, barbaloin, aloe-emodin, hydroxychromones, and aloesone [68]. *Areca catechu* L. is locally known as Lugus. This plant’s seeds are mashed, and the juice is applied to cuts and scabies. The seeds can also be utilized to fight intestinal parasites and lower blood pressure and heart rate. It is used as a relaxant when eaten with Piper betle leaves. It contains a little amount of nicotine, [69]. *Brucea javanica* Merr. is locally known as Gompoit and used to cure stomachache, malaria, dandruff, and parasites (lice and worms) [68]. *Capsicum frutescens* L. is locally known as Ladoh. The leaves and fruits of this plant, which contain alkaloids and essential oils [70], are crushed and used to treat skin and common ailments in pregnancy. *Mallotus miquellianus* is used to treat jaundice, diarrhea, fever, and itchy skin [71]. *Psidium guajava* L. is locally known as Liaba or Siabas. The young leaves are used to cure stomachache, diarrhea, dysentery, and acute gastroenteritis. The fruit of the plant possesses the glucosides, guaijaverin, crataegolic, luteioic, and argamolic acids [72].

### 4.2. Malaysian Hepatoprotective Plants

**Figure 2:**
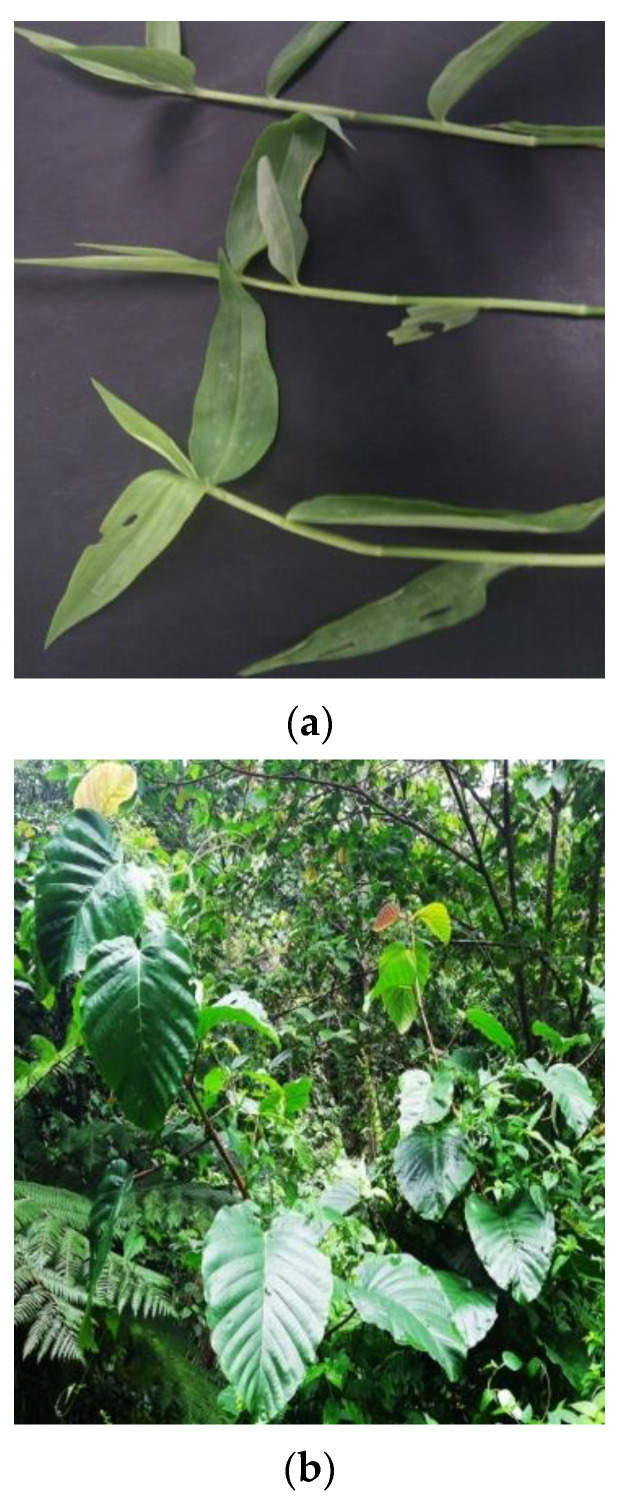

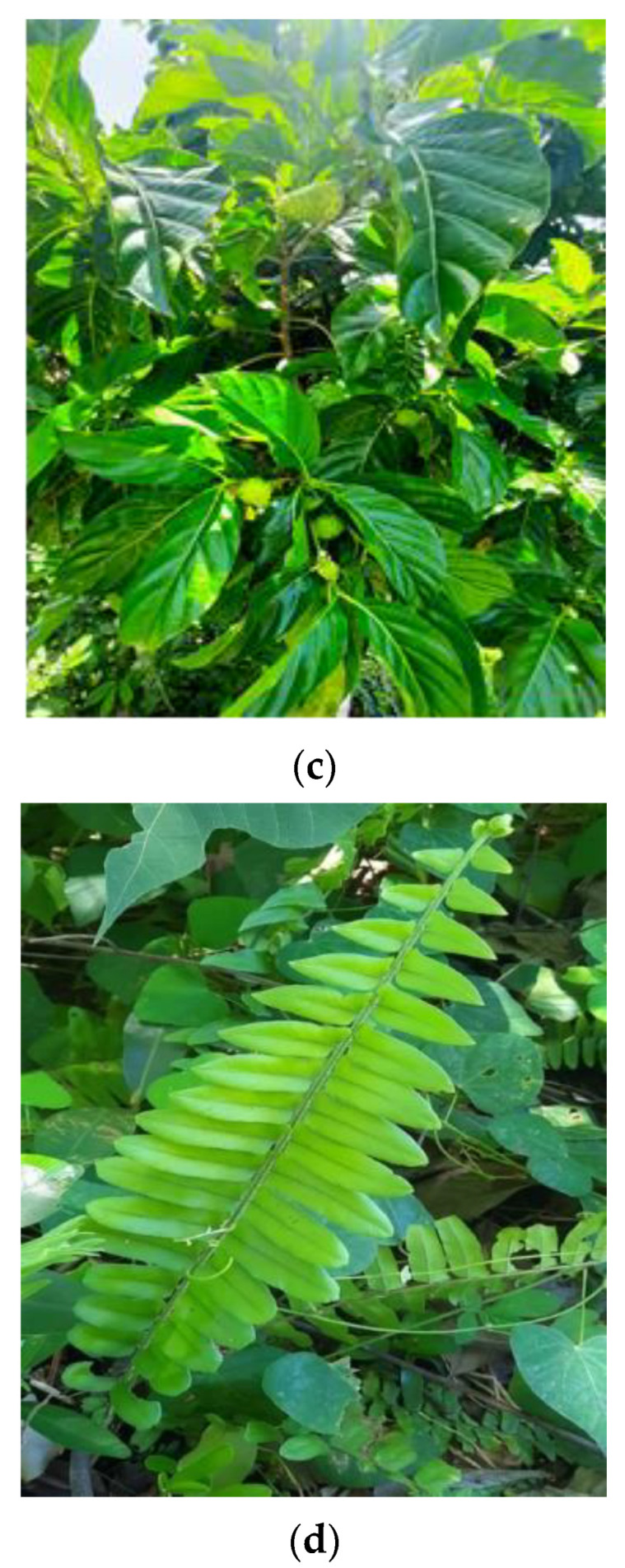
(**a**) *Commelina Nudiflora*: its leaves measure 7–10 cm in length and 1–2.5 cm in width, and its stalks are 35–60 cm in length. (**b**) *Dillenia suffruticosa*: it is usually 4–10 m tall with alternate, oval, penniveined, and serrated leaves. The young leaves are reddish, whereas the mature ones are dark green. (**c**) *Morinda citrifolia*: it reaches a height of 3–10 m at maturity and has light green, four-angled twigs with opposite, pinnately veined, and glossy leaves attached by stout petioles. (**d**) *Nephrolepis biserrata*: it is a tropical evergreen fern with pinnate, bipinnate, and palmate leaves and its leaves are usually 5–10 cm long and 2–5 cm wide and stalk range from 60 to 90 cm in length.

Medicinal plants are commonly used for treating hepatotoxicity and other diseases because they are effective, cheap, and safe. Poor lifestyle, excessive alcohol consumption, and drug habits are factors that contribute to hepatic damage [73,74,75]. Modern chemical therapies are still not well known for the treatment of various hepatic injuries, and only a few drugs are available in this regard [73]. Therefore, many folk remedies of plant origin have been examined for their potential anti-inflammatory, antioxidant, and hepatoprotective properties [49,50,51,76]. Medicinal plants, such as Commelina nudiflora, Nephrolepis biserrata, Dillenia suffruticosa, Azadirachta indica, and Morinda citrifolia (Figure 2), are vital sources of bioactive compounds with antioxidant potential [11,77,78,79,80]. These compounds play key roles in the detoxification and removal of free radicals [47,48,49,51]. The consumption of different parts (leaves, fruits, stem, and roots) of medicinal plants with potential antioxidant properties can protect against diseases caused by oxidative stress [73,81,82,83].

Some of the Malaysian medicinal plants with hepatoprotective activity are discussed.

#### 4.2.1. *Andrographis paniculata* (Acanthaceae)

*Andrographis paniculata* is known as the “King of the Bitters”. The ethanol extract of the aerial parts of the plant has been reported to have significant antioxidant and hepatoprotective activities. The pretreatment of the plant extract at a concentration of 300 mg/kg b.w. against CCl_4_ challenged rats resulted in a 75 and 14.5% restoration of hepatic enzymes, Alanine aminotransferase (ALT), and Aspartate aminotransferase (AST). The exposure of animals to *A. paniculata* extract decreased the MDA level by 40% and increased the GSH level by 46%. The activities of CAT, GPX, QR, GST, GR, and G6PD in the hepatic tissues were significantly restored by 22 to 94%. Further, the exposure of the solvent extract of the plant reduced degenerative changes such as fatty changes, cellular hypertrophy, necrotic cells, inflammatory cell infiltration, and sinusoidal dilatation induced by CCl_4_ administration [84].

#### 4.2.2. *Bauhinia purpurea* (Leguminosae)

The plant is known as “pokok tapak kerbau” in Malay. The plant has been reported with antioxidant activity (61%), at a concentration of 200 μg/mL using a 2,2-diphenyl-1-picrylhydrazyl (DPPH) radical scavenging assay applying ascorbic acid as a standard and total phenolic content of (194.35 mg GAE/100 g) using gallic acid as a standard. The methanol extract of the plant has been reported to have hepatoprotective properties. The plant extract has been administered at a dose of 50 to 500 mg/kg b.w. for 7 days, followed by a hepatotoxicity induction using paracetamol (PCM) in rats. Exposure of *B. purpurea* extracts at a concentration of 500 mg/kg b.w. restored the activity of ALT (49%), AST (42%), and ALT (22%) in the PCM-treated group compared to the solely PCM-treated group. Histopathological alterations such as necrosis, inflammation, and haemorrhage have also been reduced by the solvent extract of the plant in PCM-treated groups [85].

#### 4.2.3. *Commelina nudiflora* (Commelinaceae)

The methanol leaf extract of the plant has been reported to scavenge 2,2-diphenyl-2-picrylhydrazyl-free radicals effectively (66% at a concentration of 500 μg/mL). Sprague Dawley rats were orally exposed to *C. nudiflora* (450 mg/kg b.w.) once daily for 14 days, followed by two doses of CCl_4_ (1 mL/kg b.w.). The effects of CCl_4_ toxicity on serum indicators of liver damage, AST and ALT, were dramatically reduced by 63% and 40% with the administration of *C. nudiflora*. The solvent extract of the plant improved the enhanced hepatic production of MDA (50%) caused by CCl_4_ in rats by increasing antioxidant levels of hepatic glutathione (GSH) and antioxidant enzymes. According to histopathological analysis, *C. nudiflora* extract protected the liver from the toxic effects of CCl_4_ and cured necrosis, hepatocyte injury as an irregular lamellar organization, dilations in the endoplasmic reticulum, fatty degeneration, and other lesions. According to immunohistochemistry analysis, pretreatment of *C. nudiflora* reduced the development of 8-hydroxy-2-deoxyguanosine (8-OHdG) and 4-hydroxy-2-nonenal (HNE)-modified protein adducts. Overexpression of the proinflammatory cytokines TNF-α, prostaglandin E2, and IL-6 were also reduced [11].

#### 4.2.4. *Clidemia hirta* (Melastomataceae)

A tropical shrub, widely distributed in the Southeast. *C. hirta’s* hepatoprotective effects and antioxidative potential have been investigated against CCl_4_-induced injuries and oxidative damage in mice. The mice were exposed to an aqueous extract of *C. hirta* at a concentration of 600 mg/kg b.w. for 14 days before receiving two doses of CCl_4_ (1.0 mL/kg b.w.) orally on days 14 and 15. Hepatic damage includes the escalation of ALT and AST (77% and 76%), MDA (38%), depletion of GSH (48%), and reduced antioxidant enzymes, CAT (34%), GPX (129%), GR (127%), GST (36%), and QR (38%), were significantly ameliorated by the administration of mice with *C. hirta* extract. Histopathological observations indicated that the pretreatment of *C. hirta* showed reduced hepatic lesions, necrosis, and fatty alterations. Furthermore, well-developed nucleated hepatocytes organized around the central vein and well-formed sinusoidal arrays were also noticed [13].

#### 4.2.5. *Curcuma xanthorrhiza* (Zingiberaceae)

The plant has been used in folk medicine for the treatment of hepatitis and other liver illnesses. The antioxidant and hepatoprotective effects have been studied. In the hexane fraction of the plant extract, total phenolic and flavonoid contents were estimated to be 61 mg GAE/g and 92 mg CE/g. The administration of hexane fraction of *C.* *xanthorrhiza* rhizome ethanol extract at a concentration of 500 mg/kg b.w. for 7 days successively reduced the levels of ALT, AST, ALP, triglyceride, and TP by 40–80%, respectively. Pretreatment of *C.* *xanthorrhiza* hexane fraction at a concentration of 500 mg/kg effectively reduced massive necrosis formation, distortion of hepatocytes, hepatocytes’ ballooning, clear cell foci formation, shrinkage of the nucleus, loss of cellular boundaries, and reticular fibers in CCl_4_-intoxicated rats’ liver section [86].

#### 4.2.6. *Cymbopogon citratus* (Gramineae)

Locally, the plant is known as “lemongrass or serai”. The stem methanol extract of *C. citratus* has been reported to have hepatoprotection against CCl_4_ intoxicated rats. For 2 weeks, rats were given *C. citratus* extract orally (100, 200, and 300 mg/kg b.w.) before being given CCl_4_ (1.2 mL/kg b.w.) on the 13th and 14th days. Hepatoprotection has been noticed in a dose-dependent manner. At the maximum concentration (300 mg/kg b.w.) of the extracts, biochemical parameters, ALT, AST and lactate dehydrogenase (LDH) were restored by 90, 79 and 28%, MDA level by 44%, reduced GSH by 48%, antioxidant enzymes, CAT, GPX, QR, GST, GR, G6PD (glucose-6-phosphate dehydrogenase:) and (GGT) Gamma-Glutamyl transferase by 19, 23, 139, 11, 29 and 2.2%. Degenerative alterations such as fatty change, necrotic cells, cellular hypertrophy, sinusoidal dilatation, haemorrhage, and inflammatory cell infiltration were significantly reduced by the pretreatment of the plant extract in the CCl_4_-treated group [87].

#### 4.2.7. *Clitoria ternatea* (Fabaceae)

The solvent extract of *C. ternatea* has shown hepatoprotective and antioxidant properties against PCM-induced hepatic damage in mice. At a concentration of 1 mg/mL, the antioxidant activity of *C. ternatea* leaf extract was 67%, and total phenolic and flavonoid contents were found to be 358 mg GAE/g and 123 mg CE/g. The PCM-induced liver toxicity trials revealed that mice treated with *C. ternatea* methanol leave extract (200 mg/kg) significantly lowered levels of ALT (61%), AST (60%), and bilirubin (73%), all of which were significantly higher in the sole PCM-treated group. The administration of *C. ternatea* leaf extract has also been shown to protect against histopathological alterations [88].

#### 4.2.8. *Dillenia suffruticosa* (Dilleniaceae)

The plant is known as “Simpoh air” or “Simpoh ayer” in Malay [89]. For 14 days, Sprague Dawley rats were exposed to methanol extract of *D. suffruticosa* leaves (200, 300, and 400 mg/kg b.w.) once daily, followed by two doses of CCl_4_ (1.0 mL/kg b.w.). In CCl_4_-intoxicated rats, *D. suffruticosa* significantly reduced the extent of MDA formation by 13% to 79%, increased reduced glutathione levels by 5% to 21%, and increased antioxidant enzyme activities by 0.43% to 35%. The histopathological analysis indicated that the plant extract protected the liver from harmful effects*,* such as fatty degeneration, necrosis, and inflammation. Moreover, transmission electron microscopy observations indicated that D. suffruticosa also reduced hepatocyte damage such as abnormal lamellar organization and endoplasmic reticulum dilations [90,91].

#### 4.2.9. *Dicranopteris linearis* (Gleicheniaceae)

The antioxidative and hepatoprotective effects of the leaf methanol extract of D. linearis against CCl_4_-induced hepatic damage in rats have been reported. The antioxidant activity of plant extract and total phenolic contents were found to be high. At a concentration of 500 mg/kg b.w. serum biochemical parameters, ALT, AST, and ALP recovered by 46–59% compared to solely CCl_4_-treated rats. Histopathological analysis of hepatic tissues in groups pretreated with D. linearis showed mild necrosis and inflammation of the hepatocytes compared to the negative control group [92].

#### 4.2.10. *Elaeis guineensis* (Arecaceae)

The hepatoprotective effect of the medicinal plant has been reported in mice. Hepatic damage was induced in mice by the administration of PCM (1 g/kg b.w.) followed by the extract treatment after 3 h for 7 consecutive days. The data indicated that mice exposed to *E. guineensis* leave extract (200 mg/kg b.w.) significantly lowered ALT (55%), AST (60%), and bilirubin (66%) levels, compared to the PCM-treated group [93].

#### 4.2.11. *Flagellaria indica* (Flagellariaceae)

The aqueous extract of *F. indica* has been found to have antioxidant and hepatoprotective properties against CCl_4_-induced hepatic damage in rats. At a concentration of 400 µg/mL, the antioxidant activity of *F. indica* leaf extract was 50%, and total phenolic and flavonoid contents were estimated to be 65 mg GAE/g and 21 mg CE/g. Adult Sprague Dawley rats were exposed to an aqueous extract of *F. indica* leaves once daily for 14 days at a concentration of 300, to 500 mg/kg b.w. before receiving a CCl_4_ dosage (1.0 mL/kg b.w.) on the 13th and 14th days. The biochemical investigations indicated the aqueous extract of *F. indica* was able to prevent the increase in AST and ALT (38–74%), as well as MDA generation (25–87%) in a dose-dependent pattern. Histopathological analysis displayed that the pretreatment of the *F. indica* extract markedly ameliorated infiltration, massive lymphocytic, sinusoidal dilation, heavy loss of cellular boundaries, and ballooning degeneration, compared to the solely CCL_4_-treated group. Transmission electron microscope ultrastructural observations showed that retreatment of the extract prevented the organelles from damage such as, loss of glycogen granules, dilated mitochondria, shrinkage of the nucleus, degenerated rough endoplasmic reticulum, and loss of granules. Furthermore, immunohistochemical observation showed that oxidative stress markers HNE and 8OHdG and pro-inflammatory markers (TNF-α, IL-6, and prostaglandin E2) were also suppressed in a dose-dependent pattern [94].

#### 4.2.12. *Lygodium microphyllum* (Lygodiaceae)

At a dosage of 65 µg/mL, the aqueous extract of *L. microphyllum* was able to scavenge DPPH radicals up to 50%. Total phenolic and flavonoid contents were found to be 206 mg GAE/g and 21 mg CE/g. The hepatoprotection of the plant extract at various concentrations (200, 400, and 600 mg/kg b.w.) against CCl_4_-induced hepatic damage has been reported. In a dose-dependent manner, *L. microphyllum* declined the increment in levels of ALT, AST, and hepatic MDA production. Immunohistochemical results indicated that production of 8-OHdG and HNE was markedly ameliorated by *L. microphyllum* pretreatment compared to the CCl_4_-treated model group. The histopathological observation of liver sections of rats exposed to *L. microphyllum* showed a reduction in hepatocellular degeneration, heavy lymphocytic infiltration, deformation of the central vein, and dilated sinusoidal spaces in a dose-dependent pattern. Ultrastructural investigations using a transmission electron microscope revealed recovery of mitochondria, derangement of the nuclear envelope with nucleus shrinkage, degraded rough endoplasmic reticulum and loss of granules in the CCL_4_-intoxicated group exposed to *L. microphyllum* in a dose-dependent manner [61].

#### 4.2.13. *Muntingia calabura* (Muntingiaceae)

Aqueous partition of methanol extract of *M. calabura* leaves (250 mg/kg b.w.) has been shown to have hepatoprotective properties against PCM intoxication. At a concentration of 27 µg/mL the extract was able to scavenge DPPH radicals up to 50%. Total phenolic and flavonoid contents were estimated to be 413 mg GAE/100 g and 21 mg CE/g. Administration of the extract reversed the effect of PCM on levels of ALT, AST, and ALP by 85, 82, and 33%, as well as the activity of SOD and CAT by 173 and 68%. The absence of necrosis and haemorrhage was confirmed by microscopic examination and histological scoring of rat liver tissue pretreated with the aqueous partition of methanol extract of *M. calabura* leaves (250 mg/kg) and followed by the oral exposure of PCM [95].

#### 4.2.14. *Melastoma malabathricum* (Melastomataceae)

The hepatoprotective activity of methanol leaf extract of M. malabathricum leaves has been investigated against CCl_4_-intoxicated rats. The rats exposed to 500 mg/kg b.w. of extracts for 7 days, followed by the induction of hepatotoxicity using CCl_4_, restored the function of ALT and AST by 49 and 80%. The histopathological observation indicated that the pretreatment with 500 mg/kg of plant extracts reduced the infiltration of leukocytes, haemorrhage, and microvesicles of steatosis compared to the CCl_4_-treated group [96].

#### 4.2.15. *Morinda citrifolia* (Rubiaceae)

The therapeutic effects of *Morinda citrifolia* ethanol leaf extract have been reported in rats fed with thermoxidized palm oil. Obesity, an increase in the oxidative stress marker, MDA, diffuse microvesicular steatosis, and mitochondrial dysfunction were all seen in thermoxidized palm oil-fed rats. The solvent extract of *M. citrifolia* prevented hepatic steatosis, increased the hepatic antioxidant enzymes SOD (66%) and GPx (62%), reduced MDA (9%), prevented mitochondrial damage, and retained normal hepatic histology and ultrastructure [80].

#### 4.2.16. *Nephrolepis biserrata* (Nephrolepidaceae)

The oral administration of *N. biserrata* extract (at doses of 125, 250, and 375 mg/kg b.w.) against CCl_4_-induced hepatic damage rats significantly depleted the elevation of enzymatic levels of ALT and AST (20–93%), reduced the extent of MDA (47–90%), increased the level of reduced glutathione (25–39%), and elevated the activities of CAT, GR, GPx, G6PD, GST, and QR (5–34%). Furthermore, the histopathological results also showed that solvent extract of the plant-reduced necrosis and fatty degeneration in CCl_4_-administered rats [97].

#### 4.2.17. *Orthosiphon stamineus* (Lamiaceae) and *Phyllanthus niruri* (Phyllanthaceae)

The ethanol extracts of *O. stamineus* and *P. niruri* have been reported to have hepatoprotection against thioacetamide-intoxicated rats. Administration of *O. stamineus* (200 mg/kg b.w.) significantly restored ALT (52%), AST (38%), and MDA (45%) levels in thioacetamide-induced rats. Similarly, *P. niruri* (200 mg/kg b.w.) exposure to thioacetamide (TAA, 200 mg/kg b.w.)-treated rats regulated the expression of growth factor (TGFβ), collagenα1 (Collα1), matrix metalloproteinase-2 (MMP2), and tissue inhibitor of matrix metalloproteinase-1 (TIMP1) genes compared to only the thioacetamide-treated group [98,99].

#### 4.2.18. *Synedrella nodiflora* (Asteraceae)

The aqueous leaf extract of *S. nodiflora* has been reported to provide hepatoprotection against CCl_4_-mediated hepatic injury in rats. The oral administration of *S. nodiflora* (300 mg/kg b.w.) for 2 weeks, followed by the administration of CCl_4,_ significantly restored the levels of ALT (20%), AST (41%), GSH (44%), and MDA (50%). Pretreatment of *S. nodiflora* extract markedly enhanced the hepatocellular arrangements with only minimal lymphocytic infiltration and fatty changes compared to the CCl_4_-intoxicated group. Furthermore, the ultrastructural observation indicated that the exposure of *S. nodiflora* prevented the organelles from damage, with well-protected cellular boundaries [100].

### 4.3. Phytochemical Compounds of Malaysia Hepatoprotective Plants

The phytochemical compounds have been noticed in Malaysian hepatoprotective plants. Some of them are tabulated (Table 1).

The above-mentioned phytochemical ingredients, including alkaloids, saponins, flavonoids, steroids, anthraquinones, etc., have been reported to have hepatoprotective potential [114,115,116,117,118,119].

### 4.4. Chromatographic Analyses of the Active Constituents of Malaysian Hepatoprotecive Plants

The results from the high-pressure liquid chromatography, gas chromatography, and mass spectrometry of the active constituents of the hepatoprotective plants are presented in Table 2.

Chromatographically analyzed active constituents*,* including andrographolide (diterpenoid) [133], lutein [134], eugenol (phenol) [135], phytol (diterpene alcohol) [136], fucosterol (sterol) [137], quercetin (flavonoid) [138], squalene (triterpene) [139], gamolenic acid (fatty acid, also known as gamma-linolenic acid) [140], rosmarinic acid (phenolic compound) [141], stigmasterol (sterol) [142], kojic acid (pyranone) [143], linalool (monoterpenoid) [144], kaempferol (flavonoid) [145], ellagic acid (polyphenol), [146], oleanolic acid (triterpenoid) [147] etc., have been reported with antioxidant, hepatoprotective, and anti-inflammatory properties.

## 5. The Methodology of the Review

The data were obtained from various internet databases, including ScienceDirect, PubMed, ACS publications, Wiley, etc., and proceedings and theses. Records were searched with keywords related to Malaysian medicinal plants, distribution, taxonomy, hepatic protection, hepatic damage, bioactivity, biochemical composition, antioxidant, oxidative stress, etc. Around 227 records, approximately from the years 2000 to 2021, were retrieved and screened. Among these, about 80 records were excluded due to being outside the scope of the review. Finally, a total of 147 records were adopted for the present review article. Furthermore, data from organizations such as the World Health Organization were also included.

## 6. Conclusions

This review identified various plant extracts with hepatoprotective activities against harmful chemicals that trigger hepatic damage. Furthermore, this study investigated the use of medicinal plants in the folk medicine of Malaysia. These plants may be added as new alternatives to the limited therapeutic options that currently exist for treating hepatic injuries; these plants should be considered for future research. The study also highlighted different phytochemical compounds (tannins, saponins, quinones, terpenoids, steroids, flavonoids, phenols, alkaloids, glycosides, cardiac glycosides, coumarins, anthocyanins, etc.) with hepatoprotective properties found in Malaysian plants. In addition, the review showed various bioactive compounds, including andrographolide, lutein, eugenol, phytol, fucosterol, quercetin, squalene, gamolenic acid, rosmarinic acid, stigmasterol, kojic acid, linalool, kaempferol, ellagic acid, oleanolic acid, etc., with antioxidant, hepatoprotective, and anti-inflammatory properties. The potent hepatoprotective properties of the bioactive compounds obtained from natural origins represent an exciting avenue in the search for effective and cheap hepatoprotective agents, particularly at this time when there is an urgent requirement for innovative and effective drugs. Further studies on the isolation, purification, and characterization of bioactive compounds and trials using animal models need to be conducted to ascertain the safety of these compounds as good alternatives for treating hepatic disorders.

## Figures and Tables

**Table 1:** The phytochemical compounds of Malaysia hepatoprotective plants.

Name of the Plants	Phytochemical Compounds	References
*Andrographis paniculata*	Alkaloids, saponins, flavonoids, tannins, terpenoids, steroids, polyphenols, diterpenoid lactones	[84,101]
*Bauhinia purpurea*	Alkaloids, steroids, sterols, glycosides, saponins, flavonoids, tannin, phenolic, anthraquinones	[102]
*Commelina nudiflora*	Alkaloids, anthraquinones, flavonoids, phytosterol, saponins, tannins, triterpenoids	[11]
*Cymbopogon citratus*	Aldehydes, ketones, alcohols, esters	[103]
*Clitoria ternatea*	Alkaloids, tannins, glycosides, resins, steroids, saponins, flavonoids, phenols	[104]
*Curcuma xanthorrhiza*	Terpenoid, phenols, flavonoid, saponin, cardiac glycoside, alkaloid, anthraquinone, tannin	[105]
*Clidemia hirta*	Tannin, flavonoid, saponin, terpenoid	[106]
*Dicranopteris linearis*	Tannins, saponins, quinones, terpenoids, steroids, flavonoids, phenol, alkaloids, glycosides, cardiac glycosides, coumarins, anthocyanin, betacyanin	[107]
*Dillenia suffruticosa*	Alkaloids, anthraquinones, flavonoids, phytosterol, saponins, tannins, triterpenoids	[90]
*Elaeis guineensis*	Coumarins, phenolic, saponins, tannins, terpenoids, steroids, flavonoids, carbohydrate	[108]
*Flagellaria indica*	Flavonoids, tannins, saponins, steroids, triterpenoids, alkaloids, phytosterols	[94]
*Lygodium microphyllum*	Flavonols (quercetin and quercetin-3-O-glucopyranoside)	[12]
*Muntingia calabura*	Phlobatannins, reducing sugar, terpenoids, flavonoids, alkaloids, steroids	[109]
*Melastoma* *malabathricum*	Tannins, steroids, phenolic, flavonoids	[110]
*Morinda citrifolia*	Steroids, cardiac glycosides, phenol, tannins, terpenoids, alkaloids, resins, carbohydrates, flavonoids, anthraquinones, phylobatannins, reducing sugar, saponins, protein, lipids, fats	[111]
*Nephrolepis biserrata*	Alkaloids, anthraquinones, flavonoids, phytosterol, saponins, tannins, triterpenoids	[97]
*Orthosiphon stamineus*	Alkaloids, saponins, flavonoids, tannins, terpenoids, steroids	[112]
*Phyllanthus niruri*	Saponins, alkaloids, phenols, terpenoids, flavonoids	[99]
*Synedrella nodiflora*	Flavonoids, alkaloids, tannins	[113]

**Table 2:** Chromatographic analyses of the active constituents of the reviewed hepatoprotective plants.

Name of the Plants	Active Constituents	References
*Andrographis paniculata*	Apigenin-7-O-β-D-glucuronide, 5,4′-dihydroxy-7-methoxy-8-O-β-D-glucopyrarosyl-flavone, 5,4′-dihydroxy-7,8-dimethoxyflavone, 14-deoxyandrographiside, andrographolide, isoandrographolide, neoandrographolide, 14-deoxyandrographolide, dehydroandrographolide, dihydroxy dimethoxy flavone	[120]
*Bauhinia purpurea*	5,6-Dihydroxy-7-methoxyflavone 6-O-β-D-xylopyranoside, bis (3′,4′-dihydroxy-6-methoxy-7,8-furano-5′,6′-mono-methylalloxy)-5-C-5-biflavonyl, (4′-hydroxy-7-methyl 3-C-α-L-rhamnopyranosyl)-5-C-5-(4′-hydroxy-7-methyl-3-C-α-D-glucopyranosyl) bioflavonoid, bibenzyls, dibenzooxepins, phytol fatty esters, lutein, β-sitosterol, isoquercitin, astragalin	[121]
*Commelina nudiflora*	Phenol, benzyl alcohol, eugenol, phenol, 2,4-bis(1,1-dimethylethyl), dodecanoic acid, hexadecanoic acid ethyl ester palmitic acid ester, N-hexadecanoic acid, palmitic acid, phytol, diterpene alcohol, 9,12-octadecadienoic acid (Z, Z)-	[11]
*Clidemia hirta*	----	
*Curcuma xanthorrhiza*	Bisdemethoxycurcumin, demethoxycurcumin, curcumin	[122]
*Cymbopogon citratus*	Heptanal, camphene, sabinene, 6-methylhept-5-en-2-one, citronellal, geranyl acetate	[103]
*Clitoria ternatea*	Butyl-2-methyl-propylphthalate, pentadecanoic acid butyl-2-methylpropylphthalate, butyl octyl phthalate, diisononyl phthalate, lignoceric acid, dodecanoic acid, methyl ester, octadecanoic acid, methyl ester, phthalic acid, 4-cyanophenyl nonyl ester, Di-n-octyl phthalate	[123]
*Dillenia suffruticosa*	Phenol, benzyl alcohol, 2H-pyran-2-one, 4,6-dimethyl-, phenol, 2,4-bis(1,1-dimethylethyl), dodecanoic acid, hexadecanoic acid, methyl ester, n-hexadecanoic acid, phytol	[90]
*Dicranopteris linearis*	Furan, 5,5-dimethyl furan-2 (5H)-one, dodecane, 1,2,3-propanetricarboxylic acid, triethyl citrate, 1,2-benzenedicarboxylic acid, dibutyl ester, phytol, quercetin 7,3′,4′-trimethoxy, hexadecanoic acid, dioctyl ester	[107]
*Elaeis guineensis*	Linoleic acid, thianapthene-2-carbonyl chloride, cyclopropane, stigmasterol, pyridine, kojic acid, indole-2-one, pyrimidine, benzo[h] quinoline, phenol, 3,5 bis (1-1-dimethylethyl), ergost-7-en-3-ol, cholestane, γ-sitosterol, α-tocopherol, fucosterol, β-tocopherol, campesterol, palmitic acid, β-sitosterol	[124]
*Flagellaria indica*	---	
*Lygodium microphyllum*	Quercetin, stigma-5(6)-en-3β-ol, stigmast-4-en-3-one, quercetin-3-O-glucopyranoside	[12,125]
*Muntingia calabura*	Myrcene, thymol, α-terpinol, linalool, geraniol, nerol, citronellol, eugenol, α-ionone, β-sitosterol, α-amyrin, lupelol, α-tocopherol, β-carotene, fumaric acid, succinic acid, niacin, malic acid, cinnamic acid, pyridoxine, gallic acid, ascorbic acid, glucose, fructose, pantothenic acid, biotin, thiamine, kaempferol, catechin, quercetin, riboflavin, folic acid	[126]
*Melastoma malabathricum*	Ursolic acid, 2α-hydroxyursolic acid, asiatic acid, β-sitosterol 3-*O*-β-*D*-glucopyranoside, glycolipid glycerol 1,2-dilinolenyl-3-*O*-β-*D*-galactopyanoside, kaempferol, kaempferol 3-*O*-α-*L*-rhamnopyranoside, kaempferol 3-*O*-β-*D*-glucopyranoside, quercetin, ellagic acid, rhamnogalacturonan, homogalacturonan	[127,128]
*Morinda citrifolia*	Quercetin-3-O-α-L-rhamnopyranosyl-(1→6)-β-D- glucopyranoside, kaempferol-3-O-α-L-rhamnopyranosyl-(1→6)-β-D-glucopyranoside, octanoic acids, n-decanoic acid, allantoin, mannitol, glycerine, gamma-tocopherol and sorbitol	[111]
*Nephrolepis biserrata*	Butyrolactone, phenol, benzyl alcohol, phenol, 2-methoxy, 4H-pyran-4-one, 2,3-dihydro-3,5-dihydroxy-6-methyl, 2H-pyran-2-one, 4,6-dimethyl-, catechol, benzofuran, phenol, 2,4-bis(1,1-dimethylethyl), hexadecanoic acid, methyl ester, n-hexadecanoic acid, phytol, gamolenic acid, octadecanoic acid	[77]
*Orthosiphon stamineus*	Squalene, phytol, flavones, vitamin E, ergosterol, cholesterol, γ-elemene, α-ylangene, α-bulnesene, β-guaiene, Caryophyllene, β-vatirenene, 2H-pyran, 2-(7-heptadecynyloxy)tetrahydro-, n-hexadecanoic acid, hexadecanoic acid, ethyl ester, 9,12-octadecadienoic acid (Z,Z)-, ethyl 9,12,15-octadecatrienoate, 4H-1-benzopyran-4-one, 5,6,7-trimethoxy-2-(4-methoxyphenyl)-, pregn-4-en-18-oic acid, 11-(acetyloxy)-7,9,20-trihydroxy-3-oxo-, γ-lactone, (7α,11α,20R)-, stigmasterol, β-sitosterol, 4H-1-benzopyran-4-one, 2-(3,4-dimethoxyphenyl)-5,6,7-trimethoxy, α-amyrin	[129,130]
*Phyllanthus niruri*	Quercetin 3-O-hexosde, quercetin 3-sambubioside, kaempferol-3-O-, ellagic acid-O-hexoside, ferulic acid, chrysin, methyl gallate, methyl brevifolincarboxylate, ellagic acid-O-arabinoside, brevifolin, ethyl gallate, ellagic acid, coumaric acid, eriodictyol, luteolin, betulinic acid, oleanolic acid	[131]
*Synedrella nodiflora*	Caryophyllene oxide, 6,10,14-trimethyl-2-pentadecanone, methyl-(Z)-9-octadecenoate, caryophyllene, triacontane, 3,5,11,15-tetramethyl-1-hexadecen-3-ol, pentadecanal, geranylgeraniol	[132]

## Data Availability

Not applicable.
